# Sarcopenia in Senior Subjects with Hip Fracture: Study of Prevalence, Correlation with Obesity, and Ultrasonography of the Rectus Femoris Muscle

**DOI:** 10.1055/s-0045-1813001

**Published:** 2025-12-15

**Authors:** Maurício Rodrigues Miyasaki, Juliano Casonatto, Carolina Morgato de Mello Miyasaki, Bruno Leguizamón Baruki, Yano Altomar de Sá, Rodrigo Antonio Carvalho Andraus

**Affiliations:** 1 Graduate Program in Rehabilitation Science, Universidade Pitágoras UNOPAR Anhanguera, Londrina, PR, Brazil; 2 Research Group in Physiology and Physical Activity, Universidade Pitágoras UNOPAR Anhanguera, Londrina, PR, Brazil; 3 Faculdade de Medicina, Universidade Federal do Paraná, Curitiba, PR, Brazil; 4 Irmandade da Santa Casa de Londrina, Paraná, Brazil; 5 Graduate Program in Human Movement and Rehabilitation, Universidade Evangélica de Goiás (UniEVANGELICA), Anápolis, GO, Brazil

**Keywords:** hip fractures, obesity, sarcopenia, ultrasonography, fraturas do quadril, obesidade, sarcopenia, ultrassonografia

## Abstract

**Objective:**

To determine the prevalence of probable sarcopenia in senior patients with hip fractures and to evaluate the correlation between ultrasound measurements of the rectus femoris (RF) muscle, obesity, and the diagnosis of sarcopenia.

**Methods:**

The present study included 65 participants aged ≥ 60 years old admitted due to hip fractures. We administered the Strength, Assistance with Walking, Rising from a Chair, Climbing Stairs, and Falls (SARC-F) questionnaire, and measured the calf circumference and handgrip strength. We also assessed the thickness and cross-sectional area of the bilateral RF muscle using ultrasound.

**Results:**

We identified probable sarcopenia in 13 participants (20.6%). The mean RF thickness was 1.03 cm (standard deviation [SD] = 0.22) for the right thigh and 1.03 cm (SD = 0.23) for the left thigh. The mean cross-sectional area was 2.61 cm ^2^ (SD = 0.71) in the right thigh and 2.97 cm ^2^ (SD = 0.69) in the left thigh. The average calf circumference was 31 cm (SD = 4.29) for the right leg and 31 cm (SD = 4.31) for the left leg. We did not find correlations between ultrasound measurements of the RF muscle and potential sarcopenia. The diagnosis of probable sarcopenia was four times more likely in subjects who were overweight and had hip fractures.

**Conclusion:**

The prevalence of probable sarcopenia was 20.6%. There was no correlation between ultrasound measurements of the RF and the presence of sarcopenia. Overweight significantly increased fourfold the likelihood of probable sarcopenia in this population.

## Resumo

**Objetivos:**

Determinar a prevalência de provável sarcopenia em pacientes idosos com fraturas de quadril e avaliar a correlação entre as medidas ultrassonográficas do reto femoral (RF), obesidade e diagnóstico de sarcopenia.

**Métodos:**

Sessenta e cinco participantes com idade ≥ 60 anos hospitalizados com fraturas de quadril foram incluídos. O questionário SARC-F foi aplicado e a circunferência da panturrilha e a força de preensão manual foram medidas. A espessura bilateral do RF e a área de secção transversal foram avaliadas por ultrassonografia.

**Resultados:**

Provável sarcopenia foi identificada em 13 participantes (20,6%). A espessura média do RF foi de 1,03 cm (desvio padrão [DP] = 0,22) para a coxa direita e 1,03 cm (DP = 0,23) para a coxa esquerda. A área de secção transversal média foi de 2,61 cm ^2^ (DP = 0,71) na coxa direita e 2,97 cm ^2^ (DP = 0,69) na coxa esquerda. A circunferência média da panturrilha foi de 31 cm (DP = 4,29) para a perna direita e 31 cm (DP = 4,31) para a esquerda. Não foram encontradas correlações entre as medidas ultrassonográficas do RF e a sarcopenia provável. Indivíduos com sobrepeso e fratura de quadril apresentaram quatro vezes mais chances de apresentar o diagnóstico de sarcopenia provável.

**Conclusão:**

A prevalência de sarcopenia provável foi de 20,6%. Não foi observada correlação entre as medidas ultrassonográficas do RF e sarcopenia. O sobrepeso esteve significativamente associado a um aumento de quatro vezes na probabilidade de sarcopenia provável nessa população.

## Introduction

Sarcopenia in senior patients is a severe public health issue due to its relationship with an increased risk of falls and fractures, 1 to the limitation of the ability to perform daily living activities, 2 and to the association with heart and respiratory diseases and cognitive impairment. 3 Sarcopenia limits mobility by decreasing gait speed, 4 contributing to a reduced quality of life, loss of independence, and potential institutionalization requirement. 3

Sarcopenia is a significant risk factor in patients with hip fractures due to its association with the two etiological factors contributing to their occurrence, that is, osteoporosis and falls, 5 6 and its subsequent complications. Senior adults who suffer hip fractures typically face prolonged hospital stays, 7 high 1-year mortality rates, 7 and greater rehabilitation challenges, often resulting in more pronounced functional limitations.

Several publications compared surgical techniques and implant types for treating hip fractures in senior subjects, 8 with highly reliable protocols for planning surgical treatment. Clinical care and the adoption of orthogeriatric care 9 by multidisciplinary teams also ensure better surgical outcomes. However, sarcopenia, a factor significantly influencing the rehabilitation and prognosis of these senior patients, has not yet received the same attention in the management of these subjects.

The present study aimed to determine the prevalence of sarcopenia in senior patients hospitalized with hip fractures, to evaluate the correlation between ultrasound measurements of the rectus femoris muscle and the diagnosis of sarcopenia, and to verify the correlation between obesity and the diagnosis of sarcopenia.

The present study hypothesizes that the senior population with femur fractures has a high prevalence of sarcopenia, and that the ultrasonographic thickness of the rectus femoris muscle and obesity correlate with the diagnosis of sarcopenia.

## Methods

The present cross-sectional study received approval by the institution's ethics committee (opinion number 6.594.376; CAAE 7365123.0.0000.0099).

The study evaluated 65 patients aged ≥ 60 years old admitted from March 29, 2023, to March 28, 2024, with a diagnosis of proximal femur fracture. We included subjects with the cognitive capacity to answer the questionnaires and who, along with a responsible family member, agreed to participate in the study. We excluded patients with neoplastic disease, those admitted to the intensive care unit (ICU) before surgery, and those who demonstrated cognitive impairment rendering them unable to complete the self-administered SARC-F questionnaire.

We invited the patients to participate in the study and assessed them before surgery. This assessment collected data on comorbidities, fracture type, weight, and height, reported by the participants or their family members, to calculate the body mass index (BMI). Additionally, all participants completed the Strength, Assistance with Walking, Rising from a Chair, Climbing Stairs, and Falls (SARC-F) questionnaire.

**Fig. 1:**
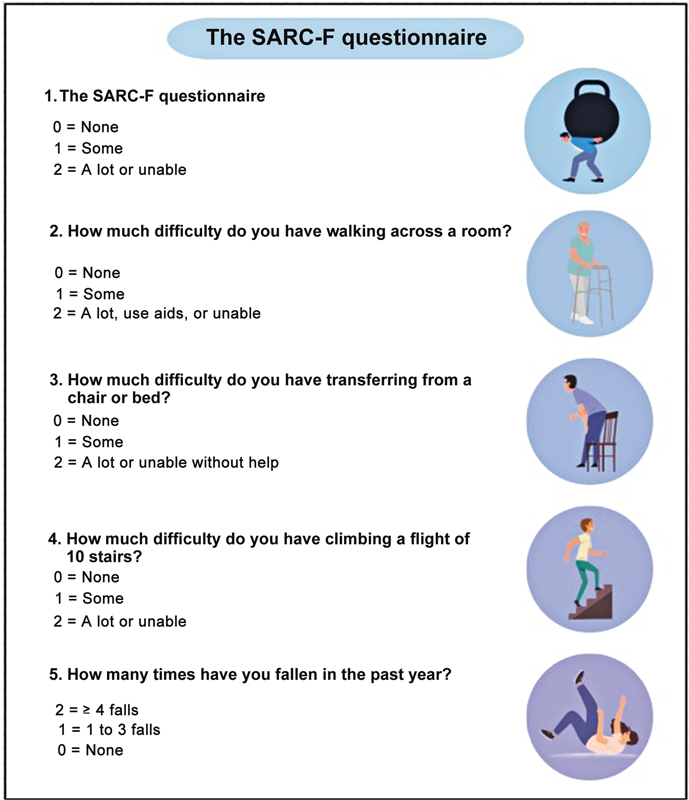
Strength, Assistance with Walking, Rising from a Chair, Climbing Stairs, and Falls (SARC-F) questionnaire.

The SARC-F ( Fig. 1 ) is a five-item self-report questionnaire designed to screen for sarcopenia risk. It assesses subjects' perceptions of their limitations in key functional areas, including muscle strength, walking ability, ability to rise from a chair, climb stairs, and history of falls. Barbosa-Silva et al. 10 validated the Portuguese-language version of the SARC-F for use in Brazil.

We performed the handgrip strength testing with a portable digital Instrutherm DM-90 (Instrutherm) device while the participant was lying down with the forearm on the dominant side supported along the body. We made 3 attempts with a 1-minute interval and recorded the highest value obtained. We measured the calf circumference 2 cm below the anterior tibial tuberosity with the participant lying down. The ultrasonographic measurement of the thickness and cross-sectional area of the bilateral rectus femoris muscle employed a Philips Lumify (Royal Philips Inc.) device and a linear transducer at a point on the thigh equidistant between the anterior superior iliac spine and the superior pole of the patella. The examiner was a single orthopedic surgeon with > 5 years of experience using ultrasound in clinical practice, blinded to the sarcopenia diagnosis.

### Sarcopenia variable

**Fig. 2:**
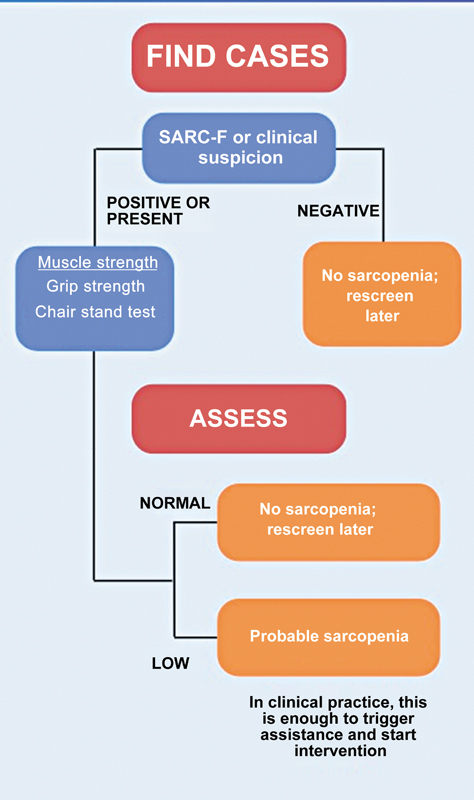
Algorithm with the initial two steps for sarcopenia diagnosis (European Working Group on Sarcopenia in Older People, EWGSOP2). *SARC-F* , Strength, Assistance with Walking, Rising from a Chair, Climbing Stairs, and Falls questionnaire.

We used the criteria of the European Working Group on Sarcopenia in Older People, revised in 2019 (EWGSOP2), 3 to determine the diagnosis of probable sarcopenia ( Fig. 2 ). This diagnosis considered a SARC-F score > 4 and palmar flexion strength < 27 kg for men and < 16 kg for women.

### Muscle mass

To estimate total muscle mass, we used the following equation developed by Lee et al.: 11 total muscle mass = 0.244 x weight + 7.80 x height + 6.6 x gender - 0.098 x age + ethnicity - 3.3. Body weight was registered in kilograms, height in meters, and age in years. This equation assigns values 0 and 1 for women and men, respectively, and - 1.2 to Asians, 1.4 to Blacks, and 0 to Caucasians. This validation of the equation for the Brazilian population employed dual-energy x-ray absorptiometry (DEXA) as the gold standard, and its correlation with the equation was R = 0.86 for men and R = 0.90 for women. The predictive value between DEXA and the equation was strong (K = 0.74; *p* < 0.001), presenting high sensitivity (89%) and specificity (86%). 11 The equation was adjusted by dividing by height squared, creating a total muscle mass index. The cutoff for identifying reduced muscle mass was ≤ 5.75 kg/m ^2^ for women and ≤ 8.50 kg/m ^2^ for men. 12

We estimated the muscle mass to investigate the correlation with the diagnosis of sarcopenia and with ultrasound measurements of the rectus femoris muscle.

### Data analysis

We performed the statistical analysis using the IBM SPSS Statistics for Windows (IBM Corp.), version 22.0, adopting a significance level of 5% ( *p* ≤ 0.05) and a 95% confidence interval (CI).

Initially, the normality of continuous variables was assessed using the Shapiro-Wilk test, as it is more appropriate for small to moderate sample sizes. Except for the left rectus femoris area, all variables presented a normal distribution, consistent with the assumptions of parametric tests. Furthermore, sample independence and variance homogeneity were observed through the Levene test, which reinforces the appropriateness of using the t-test for independent samples in comparisons between groups (normal weight versus overweight).

For the left rectus femoris area variable, which presented a nonparametric distribution, we applied the Mann-Whitney test.

We used the chi-squared test to investigate associations between categorical variables. Binary logistic regression was used to analyze the magnitude of these associations, calculating odds ratios (ORs) and their respective 95%CIs

## Results

The present study consisted of 65 participants, 22 males and 43 females. The mean age was 77 years old, ranging from 61 to 93 years old. The average BMI was 24.5 (standard deviation [SD], 5.6), with 13 participants classified as overweight (BMI ≥ 25). Thirty-eight patients presented with femoral neck fractures, and 27 had pertrochanteric fractures.

The SARC-F questionnaire score was ≥ 4 for 16 subjects, and the mean handgrip strength was 18.8 kg (SD = 7.4). Using the EWGSOP criteria, 3 we identified 13 patients with probable sarcopenia, accounting for 20.6% of the sample. Among these, 9 (69.2%) were females and 4 (30.8%) were males.

The mean thickness of the rectus femoris at ultrasound was 1.03 cm (SD = 0.22) for the right thigh and 1.3 cm (SD = 0.23) for the left thigh. The mean cross-sectional area of the rectus femoris muscle was 2.61 cm ^2^ on the right side and 2.97 cm ^2^ on the left side. The mean calf circumference was 31 cm in the right and left lower limbs. The minimum measurement was 24 cm bilaterally, and the maximum measurements were 42 cm on the right side and 44 cm on the left side. The calculation of the muscle mass index using the Lee formula yielded a mean value of 6.7 kg/m ^2^ for women and 8.8 kg/m ^2^ for men. The statistical analysis showed no correlation between the results of muscle mass index, age, calf circumference, and rectus femoris muscle measurements by ultrasound with the probable diagnosis of sarcopenia ( Table 1 ). However, we observed that subjects with probable sarcopenia had a significantly higher mean BMI ( *p* = 0.046) and lower handgrip strength ( *p* = 0.007).

**Table 1:** Comparison of anthropometric, functional, and muscular variables between senior subjects with and without probable sarcopenia after hip fracture

Variable	Probable sarcopenia	*n*	Mean value	SD	*t-value*	*P-value*
Muscle mass index (kg/m ^2^ )	No	50	19	5.6	−1.468	0.147
	Yes	13	21	6.8		
Age (years)	No	50	76	1.1	−0.856	0.395
	Yes	13	78	2.2		
BMI (kg/m ^2^ )	No	50	23	0.613	−2.040	0.046
	Yes	13	27	2.482		
Handgrip strength (kg)	No	50	20	7.3	2.770	0.007
	Yes	13	14	5.4		
Calf circumference (cm)	No	50	31	5.2	−0.190	0.850
	Yes	13	32	41.1		
Rectus femoris thickness (cm)	No	50	1.020	0.218	−0.542	0.590
	Yes	13	1.095	0.225		
Cross-sectional area of the rectus femoris (cm ^2^ )	No	50	2.569	0.672	−1.194	0.237
	Yes	13	2.832	0.841		

**Abbreviations** : BMI, body mass index; cm, centimeters; cm ^2^ , square centimeters; kg/m ^2^ , kilograms per square meter (unit of BMI and muscle mass index); n, number of participants; *P* -value, statistical significance level; SD, standard deviation; t-value, Student's t-test result for independent samples.

We also found an association between the probability of sarcopenia and excessive weight (overweight and obesity) ( Table 2 ). Logistic regression indicated the large magnitude of this association, with an OR of 4.235 (95%CI = 1.135–15.799; *p* = 0.032).

**Table 2:** Association between nutritional status and probable sarcopenia in senior subjects

		Nutritional status						
		Normal		Excessive				
		*n*	%	*n*	%	X ^2^	*P-value*	Phi
Probable sarcopenia	No	32	88.9%	17	65.4%	5.033	0.025	0.285
	Yes	4	11.1%	9	34.6%			
Total		36	100.0%	26	100.0%			

**Abbreviations** : %, percentage; n, number of participants; *P* -value, statistical significance level; Phi, phi coefficient of association to measure the strength of association between categorical variables; X ^2^ , result from the Pearson's Chi-squared test.

We found no correlation between excessive weight and handgrip strength, or ultrasound measurement of the rectus femoris area and muscle thickness. However, subjects with excessive weight had larger calf circumferences ( Table 3 ).

**Table 3:** Indicators of sarcopenia in senior patients with hip fractures: comparison between eutrophic and overweight subjects

	Nutritional status	*n*	Mean value	SD	*t-value*	*P-value*
Handgrip strength (kg)	Eutrophic	36	19.85	7.18	0.460	0.647
	Overweight	26	18.96	7.98		
Calf circumference (cm)	Eutrophic	27	29.3	3.8	−4.433	< 0.001
	Overweight	22	34.3	3.8		
Right rectus femoris thickness (cm)	Eutrophic	36	1.00	0.20	−1.461	0.149
	Overweight	26	1.08	0.24		
Left rectus femoris thickness (cm)	Eutrophic	36	1.01	0.21	−0.865	0.390
	Overweight	26	1.06	0.26		
Cross-sectional area of the right rectus femoris	Eutrophic	36	2.48	0.64	−1.959	0.055
	Overweight	26	2.83	0.75		
	Nutritional status	*n*	Median	IR	Z	*P* -value
Cross-sectional area of the left rectus femoris	Eutrophic	36	2.45	1.06	−1.405	0.160
	Overweight	26	2.80	1.04		

**Abbreviations** : IR, interquartile range; n, number of participants; SD, standard deviation; *P* -value, statistical significance level; t-value, Student's t-test result for independent samples; Z, Z-value per the Mann-Whitney test.

## Discussion

The EWGSOP2 3 sarcopenia diagnostic algorithm consists of four steps ( Fig. 1 ). The first two steps use the criteria of a low SARC-F questionnaire score and low muscle strength to establish a probable diagnosis of sarcopenia. The third step of the algorithm confirms the sarcopenia diagnosis with tests to quantify muscle mass, including body DEXA, computed tomography (CT), or magnetic resonance imaging (MRI) of the thigh. For the evaluation of senior subjects, DEXA offers significant advantages, including low radiation exposure and minimal patient cooperation. 13

As in clinical practice, for patients hospitalized for fracture treatment, these resources are rarely available to the surgeon. Therefore, we chose to stop at the second step of the algorithm and separate the sample into subjects with or without a diagnosis of probable sarcopenia. According to EWGSOP2, the identification of probable sarcopenia is enough to begin the investigation of its causes and implement clinical interventions. As such, by focusing on probable sarcopenia, we ensure the validity of the diagnosis for our study, that is, the early identification of this condition in a clinical practice setting. We believe that failure to progress to the subsequent steps of the algorithm limits the ability to classify sarcopenia severity, but not the accuracy of its initial identification, which was the focus of our research.

Although the consensus on the definition and diagnostic criteria has evolved, 14 there is still difficulty in comparing the prevalence of sarcopenia in senior subjects. The prevalence of this condition varies based on the criteria used for its characterization, cutoff points, and methods for measuring strength and muscle mass. 15

In a meta-analysis, Nascimento et al. 16 reported a 10% prevalence of sarcopenia in the general population. In another meta-analysis, Petermann-Rocha et al. 17 reported a value of 16%. Although both studies analyzed healthy senior subjects, their results showed considerable variation, particularly among articles using different diagnostic criteria. The Health, Well-being, and Aging Study (SABE, from the Portuguese *Saúde, Bem-Estar e Envelhecimento* ) study, 18 conducted with senior subjects living in the city of São Paulo, Brazil, found a sarcopenia prevalence of 16.1% for women and 14% for men. The prevalence of sarcopenia is different in groups of patients with specific conditions when compared with the general population, being reported from 18% in diabetic patients to 66% in those with esophageal cancer. 15 In the literature, the prevalence of sarcopenia in senior patients with hip fractures ranged from 17 to 37%. 19 20 We established a diagnosis of probable sarcopenia in 20.2% of our patients, which seems to be a higher rate when compared with healthy senior Brazilians and consistent with other studies with senior subjects with hip fractures.

The EWGSOP2 recommends the palmar flexion strength test with a dynamometer in subjects with lower limb fractures. This test correlates well with lower limb strength, is easy to apply clinically, is used in most publications on the subject, and has better-defined cutoff points for females and males. 3 21 Although we assessed the palmar grip strength with gender-specific cutoff points, our sample size did not allow for gender-stratified analyses of the prevalence of sarcopenia or the evaluation of associations with obesity and ultrasound.

Methods for determining muscle mass for sarcopenia diagnosis, such as MRI, DEXA, or bioimpedance, are expensive or impractical for use in hospitalized patients with acute conditions. In this context, ultrasound has been identified as a practical and accessible alternative for assessing muscle mass and quality. 22 A recent meta-analysis found moderate diagnostic accuracy for sarcopenia using ultrasound to measure the rectus femoris muscle thickness in healthy senior subjects. Data compiled from 5 studies showed a sensitivity of 72% and a specificity of 72%. 23 Ultrasound as an assessment tool for sarcopenia has been advocated in hospitalized patients with critical clinical conditions. 24 For senior subjects with hip fractures, one study found a correlation between quadriceps (rectus femoris and vastus intermedius) thickness and the diagnosis of sarcopenia. 25 In contrast to these data, we found no correlation between the rectus femoris cross-sectional area and thickness at ultrasound with the diagnosis of probable sarcopenia, BMI, handgrip strength, calf circumference, or muscle mass index. Yamada et al. 26 suggested a relationship between the measurement of muscle thickness and muscle volume, and that the assessment of echogenicity relates to muscle function. 26 However, ultrasound measures of muscle quality, such as echogenicity, assessment of fiber angles, elasticity, and vascularity, are more complex and may not be available to the surgeon who will treat the patient.

The lack of correlation may be due to limitations in the ultrasound methodology. First, there is considerable heterogeneity in the literature regarding examination protocols, particularly in patient positioning and the anatomical landmarks. 23 Another potentially influential factor is that our measurements were not made by an ultrasound specialist, as the primary objective was to evaluate the feasibility of utilizing this tool in routine clinical practice. As such, we intended to evaluate the tool in the hands of the attending physician, who, although an orthopedist with ultrasound experience, is not a radiology specialist.

Lower limb muscles are the most commonly evaluated for the ultrasound diagnosis of sarcopenia, probably because they are easier to measure and more directly associated with mobility and activities of daily living compared with the muscles of the trunk or head. 23 Furthermore, ultrasound assessment of the masseter muscle has been associated with the risk of dysphagia, while evaluation of the biceps brachii has been associated with the ability to self-feed. 25

We observed significant heterogeneity in the cited studies regarding patient characteristics, sites, and muscles examined, especially regarding the diagnostic criteria for sarcopenia, which makes comparison difficult. Although the EWGSOP2 consensus acknowledges that ultrasound demonstrates good validity for determining muscle mass, it emphasizes the need for further research to validate the method for specific clinical conditions. This caveat is largely due to challenges such as the lack of standardized cutoffs for sarcopenia diagnosis using ultrasound, the heterogeneity of examination techniques and sites (upper versus lower limbs, different muscles) across studies, and the limited correlation observed to date between ultrasound measurements and relevant clinical outcomes. These factors hinder the universal application and standardized interpretation of ultrasound for the diagnosis of sarcopenia in clinical practice.

We found a strong positive correlation between excessive weight (BMI > 24.9) and a diagnosis of probable sarcopenia. However, some confounding factors require consideration. Our analysis did not account for gender-specific variations in muscle mass decline or differences in fat distribution between ethnic groups. Moreover, obesity itself is an independent risk factor for falls and fractures in senior subjects. However, this finding makes sense when considering that we did not find significant differences in muscle strength or rectus femoris thickness between normal-weight and overweight patients. Aging is associated with a proportional increase in fat and changes in its distribution across the body, and these changes appear to present a pathogenetic link. 27

To further understand the association between excessive weight and probable sarcopenia, we characterized the demographic and functional profile of the subgroup of overweight participants diagnosed with probable sarcopenia ( *n* = 9). This group consisted of 78% women and 22% men. The mean handgrip strength was 13.4 ± 6 kg, and the mean SARC-F score was 6 ± 2 points, indicating limitation in daily activities. These data complement Table 2 , providing a more detailed overview of the clinical and functional characteristics of the overweight subjects who also had probable sarcopenia in our sample. The mean BMI was 27 ± 2 kg in sarcopenic patients and 23 ± 0.6 kg in nonsarcopenic subjects. The large difference in SD magnitude may be due to sample size.

In our study, the mean BMI of subjects with probable sarcopenia was 27.0 kg/m ^2^ , higher than the mean BMI of 23.0 kg/m ^2^ in nonsarcopenic patients. However, the greater dispersion of BMI data in the sarcopenic group is noteworthy, evidenced by an SD of 2.482, compared with an SD of 0.613 in the nonsarcopenic group. This high heterogeneity may be attributed to the phenotypic complexity of sarcopenia, since this condition can coexist with excessive weight (characterizing obesogenic sarcopenia) or occur in subjects with normal or low weight. Furthermore, the small sample size, especially in the group with probable sarcopenia ( *n* = 13), may have affected the high SD value. Smaller sample sizes are more vulnerable to the influence of extreme values and may limit the representation of populational variability.

Obesity is often associated with chronic low-grade inflammation, which can accelerate muscle breakdown and promote insulin resistance. This inflammatory state can negatively impact muscle protein synthesis, further exacerbating muscle loss. Moreover, excessive adiposity induces hormonal changes that affect muscle metabolism. Elevated levels of adipokines, such as leptin and interleukin-6, can impair muscle function and contribute to the progression of sarcopenia. 27

Obesity and sarcopenia are two conditions that, by themselves, increase functional limitations and morbidity in the senior population. The addition of a third factor associated with high morbidity and mortality, such as hip fracture, certainly increases treatment complications and deserves attention from a prevention perspective, including diet and physical activity.

Although we did not precalculate the sample size, we tried to mitigate this limitation by presenting CIs and interpreting effect sizes to enhance the transparency and robustness of the analyses. Logistic regression revealed a statistically significant association between excessive weight and a probable diagnosis of sarcopenia, with an OR of 4.235 and a 95%CI ranging from 1.135 to 15.799 ( *p* = 0.032), indicating not only statistical significance but also a clinically relevant effect size. Even though the relatively wide CIs reflect the small sample size, the findings suggest a consistent association. In addition, the difference in handgrip strength between the groups with and without probable sarcopenia was statistically significant ( *p* = 0.004), with means of 20.7 kg and 14.6 kg, respectively, corresponding to a moderate to large effect size (Cohen's d ∼ 0.89), reinforcing its functional relevance. However, we are aware that the relatively small number of participants may have limited the statistical power of the comparisons, especially those involving ultrasound measurements, increasing the risk of type II errors. Therefore, future studies with larger samples are recommended to confirm these findings and broaden the generalizability of the results. Nevertheless, these analytical strategies provide the reader with a more complete understanding of the magnitude and clinical relevance of the observed associations, despite the sample limitations.

## Conclusion

The prevalence of probable sarcopenia in our sample was 20.6%, and we found no correlation between ultrasound measurements of the rectus femoris and the diagnosis of probable sarcopenia. Subjects with hip fractures who are overweight are four times more likely to have a probable diagnosis of sarcopenia.
